# Controlling Energy Radiations of Electromagnetic Waves via Frequency Coding Metamaterials

**DOI:** 10.1002/advs.201700098

**Published:** 2017-05-26

**Authors:** Haotian Wu, Shuo Liu, Xiang Wan, Lei Zhang, Dan Wang, Lianlin Li, Tie Jun Cui

**Affiliations:** ^1^ State Key Laboratory of Millimeter Waves Southeast University Nanjing 210096 China; ^2^ School of Electronic Engineering and Computer Sciences Peking University Beijing 100871 China

**Keywords:** energy radiations, frequency coding, metamaterials, multiple controls, phase sensitivity

## Abstract

Metamaterials are artificial structures composed of subwavelength unit cells to control electromagnetic (EM) waves. The spatial coding representation of metamaterial has the ability to describe the material in a digital way. The spatial coding metamaterials are typically constructed by unit cells that have similar shapes with fixed functionality. Here, the concept of frequency coding metamaterial is proposed, which achieves different controls of EM energy radiations with a fixed spatial coding pattern when the frequency changes. In this case, not only different phase responses of the unit cells are considered, but also different phase sensitivities are also required. Due to different frequency sensitivities of unit cells, two units with the same phase response at the initial frequency may have different phase responses at higher frequency. To describe the frequency coding property of unit cell, digitalized frequency sensitivity is proposed, in which the units are encoded with digits “0” and “1” to represent the low and high phase sensitivities, respectively. By this merit, two degrees of freedom, spatial coding and frequency coding, are obtained to control the EM energy radiations by a new class of frequency‐spatial coding metamaterials. The above concepts and physical phenomena are confirmed by numerical simulations and experiments.

## 1 Introduction

Metamaterials are artificial structures composed of periodic or nonperiodic subwavelength unit cells, which have powerful abilities to tailor electromagnetic (EM) waves in unusual ways. These special materials have been described by effective media with the continuous medium parameters.^1^, ^2^, ^3^, ^4^, ^5^ The effective permittivity and permeability can be tailored to reach the values beyond possible in nature. Hence such metamaterials behave completely different from the conventional materials. In the past decade, metamaterials constructed by artificially resonant particles^6^ have been presented to manipulate the EM waves,^7^, ^8^, ^9^, ^10^, ^11^, ^12^, ^13^, ^14^ resulting in a lot of anomalous physical phenomena such as the negative refraction,^15^, ^16^, ^17^, ^18^ perfect imaging,^19^, ^20^ and invisible cloaking.^21^, ^22^, ^23^, ^24^, ^25^, ^26^, ^27^, ^28^

Recently, the concept of digital coding metamaterials has been proposed^29^, ^30^ as an alternative approach to describe metamaterials using binary codes.^29^ The digital elements in the simplest 1‐bit coding metamaterials are “0” and “1,” which have π phase difference with unity amplitude.^29^ For the 2‐bit case, the digital units are “00,” “01,” “10,” and “11,” which have the unity amplitude but different phases as 0, π/2, π, and 3π/2, respectively. Similarly, the *n*‐bit units can be obtained by dividing the phase response over 2π by 2*^n^*. Based on this concept, various functions such as wave scattering, diffusion, redirecting, and anomalous reflection have been accomplished by arranging the digital units with corresponding coding patterns. The application scope of the coding metamaterials is not only limited to microwave, but also extended to the terahertz frequencies^31^, ^32^, ^33^, ^35^ and acoustic waves.^36^

However, the above‐mentioned digital coding metamaterials are encoded in the spatial domain, and have a unique function over the working frequency band once the coding pattern is fixed. Here, we propose the concept of frequency coding metamaterials, which are encoded in the same way as the spatial coding metamaterials at the initial frequency but have another parameter to characterize the frequency domain properties. The major feature of the frequency coding metamaterials is that different phase response sensitivities over the frequency of the digital units are fully explored and utilized. For this purpose, we encode the units with digits “0” and “1” to represent the low and high phase sensitivities, respectively. Then we arrange these units to design the frequency coding metamaterials. Using the frequency digital coding, various controls to the EM energy radiations can be realized with a single digital coding metamaterial without changing the spatial coding pattern. By this merit, we have more freedom in manipulating the EM energies by a new class of frequency‐spatial coding metamaterials. We have verified these concepts and physical phenomena through a number of numerical and experimental results.

## 2 Frequency Digital Coding

For the conventional digital metamaterials, the digital units usually have the same shape, and different phase responses are achieved by varying their geometry parameters.^29^, ^31^, ^32^, ^33^, ^35^ However, the phase sensitivities of the units with similar structures are correlated to the phase responses at the initial frequency, and hence such two values are not chosen freely. To overcome this restriction, we propose the idea to design the frequency coding units by using differently shaped particles. The differently shaped particles with the same phase response at the initial frequency can experience different phase sensitivity over the frequency, which add another degree of freedom to the coding metamaterials. Owing to the different phase sensitivity of the digital units, the phase differences between two or more digital units will change dramatically as the frequency varies.

**Figure 1:**
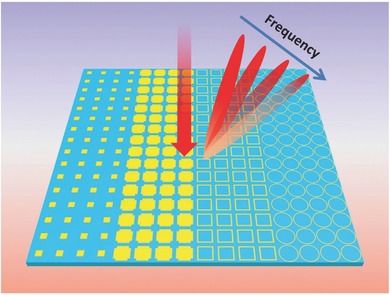
Schematic of frequency coding metamaterial illuminated by electromagnetic waves, which shows different wave‐deflection angles as the frequency changes.

Different from the conventional coding metamaterials, we need to add another frequency domain parameter, “frequency sensitivity,” to describe the digital units. For the binary case in the spatial domain, two units with the phase responses 0 and π at the initial frequency are encoded with digits “0” and “1”. While for the case in the frequency domain, another parameter is required to characterize the phase sensitivity of the units over the operational frequency band. Hence the frequency coding units can be encoded as “0–0” and “1–1,” where the first parameter describes the phase response at the initial frequency (spatial coding), while the second parameter represents the phase sensitivity level of the unit (frequency coding). For the first parameter, “0” and “1” represent 0 and π phase responses, respectively; but for the second parameter, “0” and “1” indicate low and high phase sensitivities over the frequency, respectively. The first parameter is defined in the spatial domain and describes the spatial digital coding, and thus we denote it as the spatial domain parameter; the second parameter is defined in the frequency domain and describes the frequency digital coding, and hence we denote it as the frequency domain parameter. Using the frequency digital coding, various controls to the EM energy radiations can be realized from a single coding metamaterial without changing the spatial coding pattern, as illustrated in **Figure** **1**.

The digital units of frequency coding metamaterials must be carefully designed to have the required initial phase responses and phase sensitivities to frequency, which are the key substances to the performance of the frequency coding metamaterials. To illustrate the physical mechanism behind this, we first employ the Taylor's series to express the phase response of the digital units over frequency. The phase response ϕ(*f*) over frequency *f* can be expressed as

(1) $$\begin{array}{l} \varphi \left(f\right)=\alpha_{0}+\alpha_{1}\left(f-f_{0}\right)+\alpha_{2}{\left(f-f_{0}\right)}^{2}+...+\;\;\alpha_{n}{\left(f-f_{0}\right)}^{n}\\ \;\;\;\;\;\;\;\;\;+\;\;\alpha_{n+1}{\left(f\prime \right)}^{n+1}{}^{n+1}\;\;\left(f_{0}\leq f\prime \leq f\right) \end{array}$$

where *f*_0_ is the initial frequency, α_0_ is the phase response at the initial frequency, and *α_n_* is the *n*th order of the phase response over frequency. Note that only the zeroth order of the expression has been used in the conventional coding metamaterials, in which ϕ(*f*) is simply written as

(2) $$\varphi (f)\approx \alpha_{0}$$

Here, the higher orders of the expression are ignored, indicating that the phase response is constant in the selected frequency band. Therefore, only one fixed function of EM waves can be achieved in this condition over the frequency band.

Now we propose to use the first two orders of the expression, in which the phase response is rewritten as

(3) $$\varphi (f)\approx \alpha_{0}+\alpha_{1}(f-f_{0})$$

This expression shows that the phase response of the units may vary as the frequency changes. In this case, each unit must be defined by two parameters α_0_ and α_1_, in which α_0_ is the frequency response at the initial frequency and α_1_ indicates the linear phase sensitivity over the frequency. Such two parameters required to characterize the phase property make the design of unit cells much more complicated than that in the conventional coding metamaterial. For instance, not only the phase response at the initial frequency should be designed, but also a suitable sensitivity is required at the operational frequency band. The conventional coding metamaterials with similar‐shaped particles can hardly satisfy these two requirements at the same time. Therefore, we attempt to use differently shaped particles as the unit cells to satisfy the above conditions.

## 3 Frequency‐Spatial Coding Patterns

### 3.1 Frequency Coding for 1‐Bit Spatial Coding Patterns

**Figure 2:**
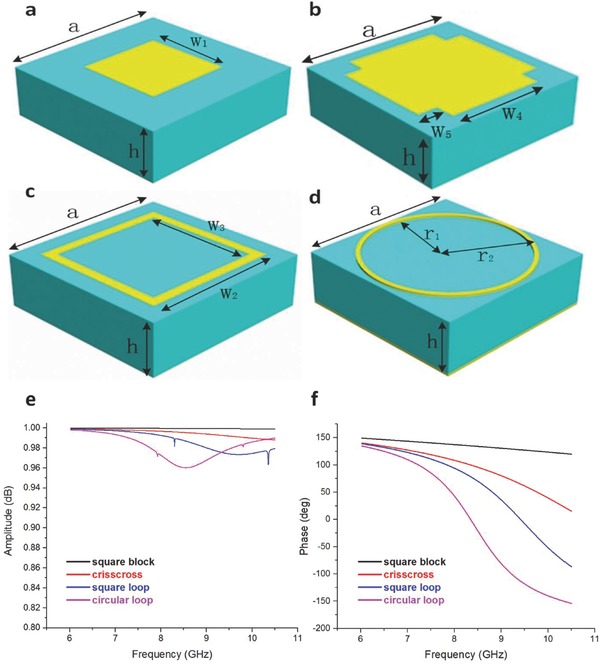
The four digital units of frquency coding metamaterials and their reflection response curves. a) Square block. b) Crisscross. c) Square loop. d) Circular loop. e) The reflection amplitude of the four digital units from 6 to 10.5 GHz. f) The reflection phase of the four digital units from 6 to 10.5 GHz.

The key point for realizing the frequency coding metamaterials is to choose the suitable unit cells. Here we use the reflection‐type metamaterials to demonstrate the concept and method. Four different subwavelength metallic structures printed on a dielectric substrate are designed to realize the frequency coding particles, as shown in **Figure** **2**a–d. The digital units are fabricated on a dielectric substrate (*ε_r_* = 2.65, loss tangent δ = 0.003) with thickness of *h* = 2 mm and period of *a* = 6 mm. The metallic patterns have different shapes: square block (Figure 2a), crisscross (Figure 2b), square loop (Figure 2c), and circular loop (Figure 2d), with the same thickness of *t* = 0.018 mm. These structures are obtained by the trial and error method using commercial software, the CST Microwave Studio. The optimized geometrical parameters are: *w*_1_ = 2 mm, *w*_2_ = 4.7 mm, *w*_3_ = 4.0 mm, *w*_4_ = 3.5 mm, *w*_5_ = 0.7 mm, *r*_1_ = 2.7 mm, and *r*_2_ = 2.85 mm. The four optimized units have similar intensity responses, as shown in Figure 2e.

To design the 1‐bit frequency coding metamaterials, we only need two units: the square block and square loop. The corresponding phase curves of the two units over the frequency band from 6 to 10.5 GHz are presented in Figure 2f, with the black line for the square block and the blue line for the square loop. The resulting phase responses of the two units are analyzed in **Table** **1**. We note that the two units have similar phase responses: ϕ_block_(*f*_0_) ≈ ϕ_loop_(*f*_0_) = 7π/9 at the initial frequency *f*_0_ = 6 GHz, and hence the spatial‐domain parameters of the two units are the same and encoded as “0”. We further observe that the two units experience different levels of phase sensitivity over the working frequency band, and the phase curves are approaching linear. In this situation, we can use the phase responses at the initial frequency and the ending frequency to determine the linear phase sensitivity parameter α_1_ approximately of the digital unit, which is expressed as

(4) $$\alpha_{1}=[\varphi (f_{1})-\varphi (f_{0})]/(f_{1}-f_{0})$$

where *f*_1_ = 10.5 GHz is the ending frequency and ϕ(*f*_1_) is the corresponding phase response of the unit. Thus the phase sensitivity parameters of the square block and square loop can be calculated as

(5) $$\alpha_{1}^{\mathrm{block}}=[\varphi^{\mathrm{block}}(f_{1})-\varphi^{\mathrm{block}}(f_{0})]/(f_{1}-f_{0})\approx -0/4.5(\mathrm{rad/GHz})$$

(6) $$\alpha_{1}^{\mathrm{loop}}=[\varphi^{\mathrm{loop}}(f_{1})-\varphi^{\mathrm{loop}}(f_{0})]/(f_{1}-f_{0})\approx -\pi /4.5(\mathrm{rad/GHz})$$

**Table 1:** Design procedure for 1‐bit digital units of frequency coding metamaterials

		Phase response over frequency (1‐bit)					
Units	Phase [deg]	Phase approximation [deg]	Spatial domain code	Phase sensitivity over frequency band [deg GHz^−1^]	Sensitivity approximation [deg GHz^−1^]	Frequency domain code	Total code
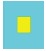	149	140	“0”	−29/4.5	0/4.5	“0”	“0–0”
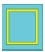	139	140	“0”	−224/4.5	−180/4.5	“1”	“0–1”

Thus we can encode α_1_^block^ as “0” and α_1_^loop^ as “1” to characterize the frequency property of the two units. Hence the overall coding states of the square block and square loop are “0–0” and “0–1,” respectively, over the operating frequency band. Once the digital units are specified, we can arrange these units with certain sequences to achieve various EM functions in the frequency domain.

**Figure 3:**
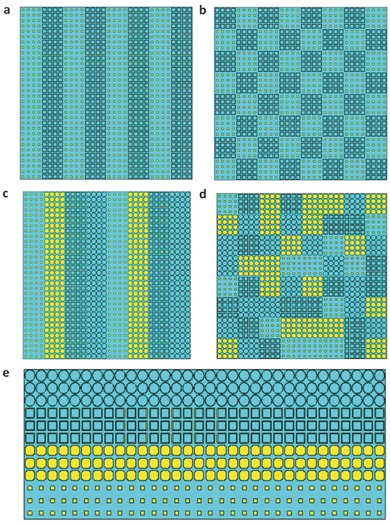
The 1‐bit and 2‐bit coding patterns of the frequency coding metamaterials. a) The pattern with 1‐bit frequency digital sequence of “0–0,” “0–1,” “0–0,” and “0–1” . b) The pattern with 1‐bit frequency digital sequence of “0–0,” “0–1,” “0–0,” “0–1”/“0–1,” “0–0,” “0–1,” and “0–0”. c) The pattern with 2‐bit frequency digital sequence of “00–00,” “00–01,” “00–10,” and “00–11”. d) The pattern with the optimized 2‐bit frequency digital sequence. e) The pattern with 2‐bit frequency digital sequence of “00–00,” “00–01,” “00–10,” and “00–11” for a period.

To illustrate the working principle of the frequency coding metamaterials quantitatively, we encoded two metamaterial examples with digital sequences of “0–0,” “0–1,” “0–0,” and “0–1” (see **Figure** **3**a) and “0–0,” “0–1,” “0–0,” “0–1”/“0–1,” “0–0,” “0–1,” and “0–0” (see Figure 3b). We adopt the super unit cell,^29^ which is composed of identical units with the size of *N* × *N* to minimize the coupling effect. Here, the size of the super unit cell is set as 4 × 4. Under the normal incidence of plane waves, the phase difference between adjacent digital units at the initial frequency *f*_0_ = 6 GHz is written as

(7) $$\varphi {\left(f_{0}\right)}^{\mathrm{relative}}=\varphi \prime \;0-1\prime (f_{0})-\varphi \prime^{}0-0\prime (f_{0})\approx 0$$

where ϕ^”0–1”^(*f*_0_) and ϕ^”0–0”^(*f*_0_) are the phase responses of units “0–1” and “0–0” at the initial frequency, and ϕ(*f*_0_)^relative^ is the phase difference between these two units. It is clear that the two digital units have nearly the same phase response. Thus the above metamaterial examples act as perfect conductors, making the incident waves be normally reflected back at the initial frequency. When the frequency increases to *f*_1_ = 10.5 GHz, the phase difference of the digital units becomes

(8) $$\varphi {\left(f_{1}\right)}^{\mathrm{relative}}=\varphi \prime \;0-1\prime \;(f_{1})-\varphi \prime \;0-0\prime \;(f_{1})\approx \pi$$

where ϕ^”0–1”^(*f*_1_) and ϕ^”0–0”^(*f*_1_) are the phase responses of units “0–1” and “0–0” at the ending frequency *f*_1_ = 10.5 GHz, and ϕ(*f*_1_)^relative^ is the phase difference between such two units. Hence the spatial coding patterns of the two examples are “0, 1, 0, 1…” with a period of *Γ*_1_ = 2 × 4 × 6 mm = 48 mm and “0, 1, 0, 1/1, 0, 1, 0…” with a period of *Γ*_2_ =$\sqrt{2}$ × 4 × 6 mm = 24 $\sqrt{2}$ mm, respectively. As a consequence, the normally incident waves will be anomalously reflected to two oblique directions at symmetrical angle θ_1_ = sin^−1^(λ/*Γ*_1_) = 36.6° for the first example, and four oblique directions at symmetrical angle θ_2_ = sin^−1^ (λ/*Γ*_2_) = 57.3° for the second example.^29^ These angles are calculated by the generalized Snell's law,^37^ where λ and *Γ* represent the free‐space wavelength (28.6 mm at 10.5 GHz) and the physical length of one period of the coding sequence.

The energy radiation patterns of these two examples should gradually change from the initial state to the ending state as the frequency increases due to the approximate linearity of the phase curves. We take the first sample (Figure 3a) as an example to quantitatively illustrate the above physical phenomenon. First, the anomalous reflection angle θ = sin^−1^(λ/*Γ*_1_) gradually decreases because *Γ*_1_ = 2 × 4 × 6 mm = 48 mm is constant and λ reduces as the frequency increases. The magnitude of anomalous reflection is small since the phase distribution of the sample 7π/9, 7π/9–2π(*f − f*_0_)/9, 7π/9, 7π/9–2π (*f − f*_0_)/9 is not uniform. That is to say

(9) $$(\varphi \prime^{}{}^{0-0}\prime^{}-\varphi \prime^{0-1}\prime )-(\varphi \prime^{0-1}\prime -\varphi \prime^{0-0}\prime )=4\pi /9(f-f_{0})\neq 0$$

in the operational frequency band except at *f*_0_ = 6 GHz and *f*_1_ = 10.5 GHz, which causes small side lobes and reduction of the anomalous reflection. At the same time, the reflection beam pointing at the *z*‐axis would almost vanish when the frequency approaches *f*_1_ = 10.5 GHz. To verify this theory, we assume that the coding sequence has infinitely large period along the *y*‐direction, making it a 1D problem. Then we calculate |*E*(*r*)|^2^ in the far distance to represent the far field energy. The electric field at the far distance can be written as

(10) $$E(r)=\int E(x)e^{j\omega t}\times e^{-j\frac{2\pi }{\lambda }r}dx$$

where *E*(*x*) is the electric field distribution of metamaterial and *r* is the distance between the observation point to the metamaterial. The analytical results show that the far‐field energy at angle θ = 0 produced by the metamaterial sample is proportional to |1+ *e^jϕ^*|^2^, where ϕ = ϕ^“0–1”^ − ϕ^“0–0”^ is the function of frequency. The detailed analysis is illustrated in the Supporting Information. The phase difference ϕ increases from 0 to π in the operational frequency band, and thus |1+ *e^jϕ^*|^2^ approaches zero when the frequency is close to *f*_1_ = 10.5 GHz. Hence the far‐field energy pointing at the angle θ = 0 would become very small.

**Figure 4:**
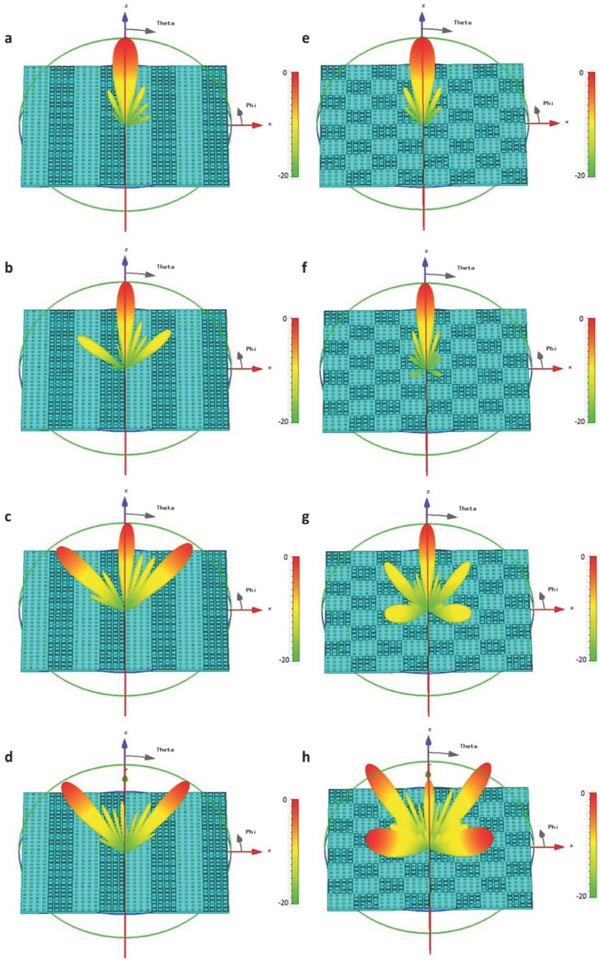
Simulated scattering patterns for the 1‐bit frequency coding metamaterials. a–d) The far‐field scattering patterns of the metamaterial encoded with 1‐bit frequency digital sequence of “0–0,” “0–1,” “0–0,” and “0–1” at 6, 8.5, 9.5, and 10.5 GHz, respectively. e–h) The far‐field scattering patterns of the metamaterial encoded with 1‐bit frequency digital sequence of “0–0,” “0–1,” “0–0,” “0–1”/“0–1,” “0–0,” “0–1,” and “0–0” at 6, 8.5, 9.5, and 10.5 GHz, respectively.

For verification, the above designs are simulated using the open boundary conditions and plane‐wave excitations in commercial software, CST Microwave Studio. For the first design, the evolution of the far‐field energy patterns with the increase of frequency is illustrated in **Figure** **4**a–d. We note that the main energy is directed to the *z*‐axis at *f*_0_ = 6 GHz (see Figure 4a) and gradually converts to two symmetrical beams as the frequency increases (see Figure 4b,c). When the frequency reaches *f*_1_ = 10.5 GHz, the original main lobe almost disappears and two symmetrical beams pointing at θ = 36° to the *z*‐axis are generated, as shown in Figure 4d. For the second design, the evolution of far‐field scattering patterns is presented in Figure 4e–h, in which the main lobe pointing to the *z*‐axis at *f*_0_ = 6 GHz is gradually changed to four symmetrical beams pointing at θ = 56° to the *z*‐axis as the frequency increases.

### 3.2 Frequency Coding for 2‐Bit Spatial Coding Patterns

We further introduce the frequency coding for 2‐bit spatial coding metamaterials, which can provide more flexibility in manipulating the electromagnetic waves. The digital unit cells of the 2‐bit frequency coding metamaterials are square block, crisscross, square loop, and circular loop, as shown in Figure 2a–d. The corresponding phase curves over frequency of such units are illustrated in Figure 2e, and the analyzed results are presented in **Table** **2**. Notice that the four units have similar phase responses at the initial frequency *f*_0_ = 6 GHz

(11) $$\varphi^{s.\mathrm{block}}(f_{0})\approx \varphi^{c.\mathrm{cross}}(f_{0})\approx \varphi^{s.\mathrm{loop}}(f_{0})\approx \varphi^{c.\mathrm{loop}}(f_{0})\approx 7\pi /9$$

where s.block represents the square block, c.cross represents the crisscross, s.loop represents the square loop, and c.loop represents the circular loop. Hence the spatial domain parameters of the four units are nearly the same, and can be encoded as “0”. However, the four units experience different levels of phase sensitivities over the working frequency band, as shown in Figure 2e. From Equation (4), the phase sensitivity parameter α_1_ is obtained as

$$\begin{array}{l} \alpha_{1}^{s.\mathrm{block}}=[\varphi^{s.\mathrm{block}}(f_{1})-\varphi^{s.\mathrm{block}}(f_{0})]/(f_{1}-f_{0})\\ \;\;\;\;\;\;\;\;\;\approx -0/9(\mathrm{rad/GHz}) \end{array}$$

$$\begin{array}{l} \alpha_{1}^{c.\mathrm{cross}}=[\varphi^{c.\mathrm{cross}}(f_{1})-\varphi^{c.\mathrm{cross}}(f_{0})]/(f_{1}-f_{0})\\ \;\;\;\;\;\;\;\;\approx -\pi /9(\mathrm{rad/GHz}) \end{array}$$

$$\begin{array}{l} \alpha_{1}^{s.\mathrm{loop}}=[\varphi^{s.\mathrm{loop}}(f_{1})-\varphi^{s.\mathrm{loop}}(f_{0})]/(f_{1}-f_{0})\\ \;\;\;\;\;\;\;\approx -2\pi /9(\mathrm{rad/GHz}) \end{array}$$

(12) $$\begin{array}{l} \alpha_{1}^{c.\mathrm{loop}}=[\varphi^{c.\mathrm{loop}}(f_{1})-\varphi^{c.\mathrm{loop}}(f_{0})]/\;\;(f_{1}-f_{0})\\ \;\;\;\;\;\;\;\;\approx -3\pi /9(\mathrm{rad/GHz}) \end{array}$$

Because α_1_^s.block^, α_1_^c.cross^, α_1_^s.loop^, and α_1_^c.loop^ are approximately equal to divisions of 2π/4.5 (rad/GHz), thus we can encode α_1_^s.block^ as “00,” α_1_^c.cross^ as “01,” α_1_^s.loop^ as “10,” and α_1_^c.loop^ as “11,” respectively, to realize the frequency domain descriptions of the four units. Hence the four units can be uniquely represented by the spatial domain parameter and frequency domain parameter, and their overall coding states are “00–00,” “00–01,” “00–10,” and “00–11,” respectively.

**Table 2:** Design procedure for 2‐bit digital units of frequency coding metamaterials

		Phase response over frequency (2‐bit)					
Units	Phase [deg]	Phase approximation [deg]	Spatial domain code	Phase sensitivity over frequency band [deg GHz^−1^]	Sensitivity approximation [deg GHz^−1^]	Frequency domain code	Total code
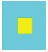	149	140	“00”	−29/4.5	0/4.5	“00”	“00–00”
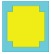	140	140	“00”	−125/4.5	−90/4.5	“01”	“00–01”
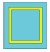	139	140	“00”	−224/4.5	−180/4.5	“10”	“00–10”
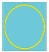	135	140	“00”	−288/4.5	−270/4.5	“11”	“00–11”

We give two metamaterial examples to demonstrate the performance of 2‐bit frequency coding. The first example is encoded by the digital sequence of “00–00, 00–01, 00–10, 00–11” (Figure 3c), while the second example is encoded by a special digital pattern (see Figure 3d) optimized for the random scattering. Under the normal incidence of plane waves, the phase differences between adjacent digital units at the initial frequency *f*_0_ = 6 GHz are

(13) $$\begin{array}{l} \varphi \prime^{}{}^{00-01}\prime (f_{0})-\varphi \prime^{00-00}\prime (f_{0})\\ \;\;\;\;\;\;\;\approx \varphi \prime^{00-10}\prime (f_{0})-\varphi \prime^{}{}^{00-01}\prime (f_{0})\\ \;\;\;\;\;\;\;\approx \varphi \prime^{00-11}\prime (f_{0})-\varphi \prime^{00-10}\prime (f_{0})\\ \;\;\;\;\;\;\;\approx \varphi \prime^{00-00}\prime (f_{0})-\varphi \prime^{00-11}\prime (f_{0})\approx 0 \end{array}$$

Thus the phase responses are approximately the same for all units, making the above two examples act as perfect conductors at the initial frequency. As a consequence, the incident wave will be reflected back in the normal direction for the two designs. Due to different phase sensitivities of the four units, when the frequency increases to *f*_1_ = 10.5 GHz, the phase responses between adjacent digital units become

(14) $$\begin{array}{l} \varphi \prime^{00-01}\prime (f_{1})-\varphi \prime^{00-00}\prime \;(f_{1})\\ \;\;\;\;\approx \varphi \prime^{00-10}\prime (f_{1})-\varphi \prime^{00-01}\prime (f_{1})\\ \;\;\;\;\approx \varphi \prime^{00-11}\prime (f_{1})-\varphi \prime^{00-10}\prime (f_{1})\\ \;\;\;\;\approx \varphi \prime^{00-00}\prime (f_{1})-\varphi \prime^{00-11}\prime (f_{1})\approx \frac{\pi }{2} \end{array}$$

Therefore, in the first example, the normally incident waves will be deflected to an oblique direction at the angle of θ = sin^−1^(λ/*Γ*_3_) = 17.3°, in which *Γ*_3_ = 4 × 4 × 6 mm = 96 mm. In the second sample, the coding pattern is optimized to redirect the electromagnetic waves to numerous directions, resulting in a significant reduction of the radar cross sections.

**Figure 5:**
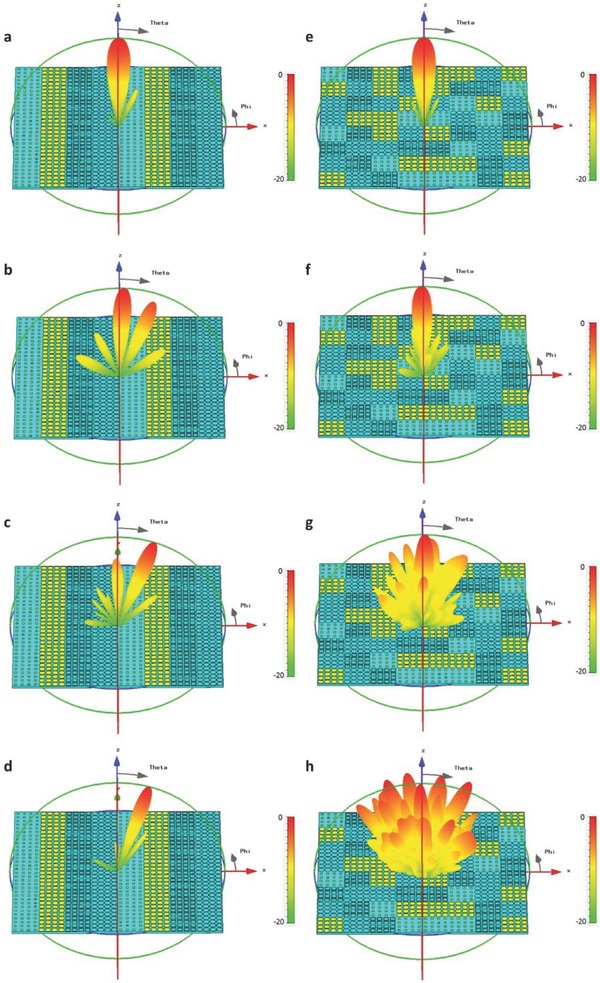
Simulated scattering patterns for 2‐bit frequency coding metamaterials. a–d) The far‐field scattering patterns of the metamaterial encoded with 2‐bit frequency digital sequence of “00–00,” “00–01,” “00–10,” and “00–11” at 6, 7.5, 9, and 10.5 GHz, respectively. e–h) The far‐field scattering pattern of the metamaterial encoded with an optimized 2‐bit frequency digital sequence at 6, 7.5, 9, and 10.5 GHz, respectively.

The above two designs are simulated using CST Microwave Studio. For the first design, the main energy points at the *z*‐axis at *f*_0_ = 6 GHz (see **Figure** **5**a) and gradually converts to a beam pointing to the right side of *z*‐axis as the frequency increases, as shown in Figure 5b,c. When the frequency reaches *f*_1_ = 10.5 GHz, the original main beam almost vanished and the side lobe evolves to a new main lobe pointing at θ = 17° to the *z*‐axis, as illustrated in Figure 5d. For the second design, the far‐field scattering patterns are illustrated in Figure 5e–h. We observe that the main lobe directing to the *z*‐axis at the initial frequency (Figure 5e) evolves to small side lobes gradually as the frequency increases (Figure 5f,g), and finally becomes numerous side lobes as the electromagnetic diffusions at the ending frequency *f*_1_ = 10.5 GHz (Figure 5h).

## 4 Nonperiodic Frequency Coding Metamaterials

From the above discussions, frequency coding metamaterials can not only be used at the initial and ending frequencies based on the spatial‐frequency encoded patterns, but also work in the whole operating frequency band with different controls of radiation EM energy. Now we discuss the nonperiodic frequency coding metamaterials. Based on Equation (9), the phase distribution of periodic frequency coding metamaterials is not uniformly distributed in the frequency band except at the initial frequency *f*_0_ and ending frequency *f*_1_. However, the nonperiodic frequency coding metamaterial has uniformly distributed phase response in the whole frequency band, in which the direction of main energy will gradually change with the frequency according to the generalized Snell's law.

**Figure 6:**
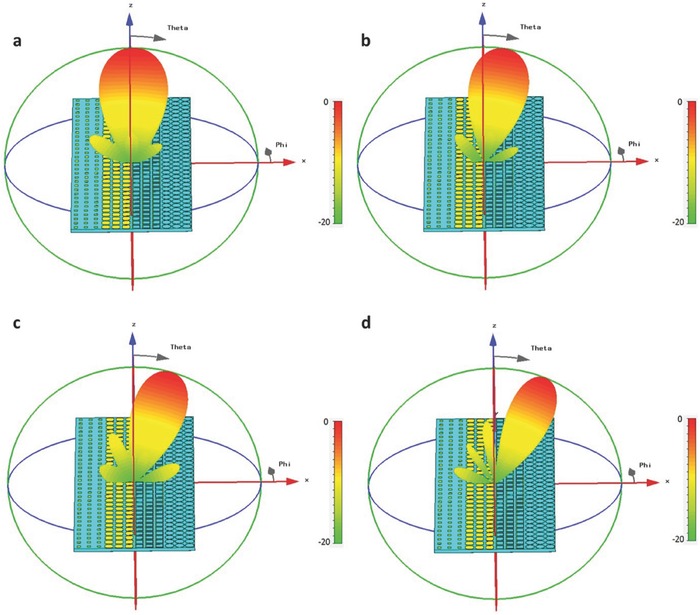
Simulated scattering patterns for the 2‐bit frequency coding metamaterial with the frequency sweeping function. a–d) The far‐field scattering patterns of the metamaterial at 6, 7.5, 8, and 10.5 GHz, respectively.

As an example, we use 2‐bit frequency digital units to construct a metamaterial sample with the coding sequence of “00–00, 00–01, 00–10, 00–11,” and each super unit cell is composed of 3 × 3 basic digital units (see Figure 3e). The spatial distributions of phase profiles of the nonperiodic frequency coding metamaterial at different frequencies are listed in **Table** **3**. Hence the normally incident plane waves will be deflected anomalously because of the changing phase gradient, and the deflected angle will gradually increase from θ_0_ = 0° to θ_1_ = 24° as the frequency changes from *f*_0_ = 6 GHz to *f*_1_ = 10.5 GHz, as clearly demonstrated in **Figure** **6**a–d. This property can be used to design frequency sweeping devices, like the reflection‐type frequency sweeping antennas.

**Table 3:** Spatial distribution of phase profile at different frequencies

	Spatial distribution of phase profile at different frequencies					
Units	6 [GHz]	7 [GHz]	7.5 [GHz]	8 [GHz]	9.5 [GHz]	10.5 [GHz]
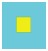	149°	143°	140°	137°	127°	120°
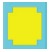	140°	128°	120°	108°	62°	15°
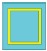	130°	123°	110°	94°	−10°	−85°
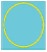	135°	110°	85°	43°	−118°	−154°

To quantitatively analyze the frequency sweeping property of the frequency coding metamaterials, the responses of digital units under the normal incidence of electromagnetic waves need to be analyzed first. We take the “00–01” unit for example, and the linear phase response over the operational frequency band can be expressed as

(15) $$\varphi (f)\prime^{00-01}\prime \approx \alpha_{0}^{\prime 00-01}\prime +\alpha_{1}^{\prime 00-01\prime }(f-f_{0})$$

where α_0_ is phase response at the initial frequency and α_1_ is calculated by Equation (4). Hence, φ(*f*)^′00 − 01′^ is further rewritten as

(16) $$\begin{array}{l} \varphi \left(f\right)\prime^{00-01}\prime \approx \alpha_{0}^{\prime 00-01\prime }+\alpha_{1}^{\prime 00-01\prime }\left(f-f_{0}\right)\\ \;\;\;\;\;\;\;\;\;=\frac{7\pi }{9}-\frac{\pi }{9}\left(f-f_{0}\right) \end{array}$$

Here, the unit of frequency *f* is in GHz. The phase responses for the other three digital units have similar expressions, and thus the phase responses of the 2‐bit digital units are

$$\varphi (f)\prime^{00-00}\prime \approx \frac{7\pi }{9}$$

$$\varphi \left(f\right)\prime^{00-01}\prime \approx \frac{7\pi }{9}-\frac{\pi }{9}\left(f-f_{0}\right)$$

$$\varphi (f)\prime^{00-10}\prime \approx \frac{7\pi }{9}-\frac{2\pi }{9}(f-f_{0})$$

(17) $$\varphi (f)\prime^{00-11}\prime \approx \frac{7\pi }{9}-\frac{3\pi }{9}(f-f_{0})$$

The analyses of the above expressions in the Supporting Information show that the anomalous deflection angle is

(18) $$\theta_{r}=\sin^{-1}\left[0.93\times \left(1-\frac{f_{0}}{f}\right)\right]$$

in which, sin^−1^(*x*) increases as *x* changes from 0 to 1, and 0.93 × (1 − *f*_0_ */f)* monotonically increases from 0 to 0.4 as the frequency changes from *f*_0_ to *f*_1_. As a consequence, *|θ_r_*| monotonically increases from 0° to 24° in the operational frequency band, which explains the above physical phenomenon.

Notice that the energy reflected by nonperiodic sample is wider than that in the periodic case, which is mainly due to the small length of the metamaterial sample in the *x*‐direction, and this problem can be alleviated by extending the size of metamaterial. In this case, the sweeping range of angle θ will be reduced due to the reduction of the phase difference over the unit length.

## 5 Conclusion

In summary, we proposed the concept of frequency coding metamaterials, which can explore the frequency‐domain property of metamaterials. The key substance to the frequency coding metamaterial is different phase sensitivities over the frequency domain of the metamaterials. We employed four differently shaped digital units to physically utilize the different sensitivity properties. The proposed method provides another degree of freedom to construct metamaterials, and opens new possibilities to control the electromagnetic waves at different frequencies. By using the 1‐bit digital units “0–0” and “0–1,” and 2‐bit digital units “00–00,” “00–01,” “00–10,” and “00–11” with predesigned coding sequences, we can manipulate electromagnetic waves in the frequency domain, which can achieve different functions over frequency (e.g., frequency sweeping) without redesigning the structure. In this manuscript, the digital units were designed to have same phase responses at the initial frequency, but it is not necessary. The digital units with different initial phase states can be further investigated to achieve more flexible different functions. The proposed concepts and methods can also be extended to the terahertz frequencies and even to the optical region.

## 6 Experimental Section

**Figure 7:**
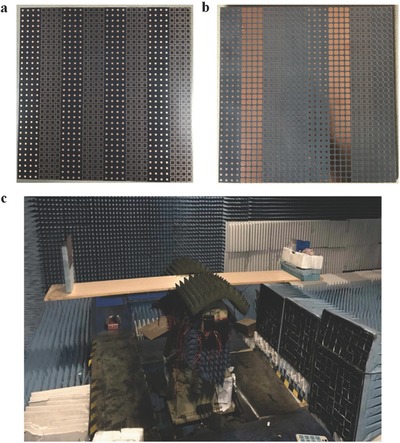
The fabricated samples of the frequency coding metamaterials and the measurement system. a) The fabricated sample of the 1‐bit frequency coding metamaterial. b) The fabricated sample of the 2‐bit frequency coding metamaterial. c) The experimental setup.

To experimentally validate the performance of the frequency coding metamaterials, two samples were fabricated, as shown in **Figure** **7**a,b, which correspond to the coding layouts given in Figure 3a,c, respectively. The metamaterials were fabricated by printing planar metallic structures on the top surface of a commercial F4B substrate with dielectric constant 2.65 and loss tangent of 0.003. Both metamaterial samples had an area of 192 × 192 mm^2^ (5.28 × 5.28λ^2^ at the central frequency 8.25 GHz) and contained 32 × 32 = 1024 digital units. Each digital unit occupied an area of 6 × 6 mm^2^ (0.165 × 0.165λ^2^). The photograph of the experimental setup for the measurement of far‐field energy patterns in the horizontal plane is illustrated in Figure 7c. A wideband horn antenna with the working bandwidth from 6 to 12 GHz was employed as the feeding antenna to generate the quasi‐plane waves for the frequency coding metamaterial. Both the feeding antenna and the sample were coaxially mounted on a supporting board at the distance of 1.8 m, and could be automatically rotated 360° in the horizontal plane with high precision.

**Figure 8:**
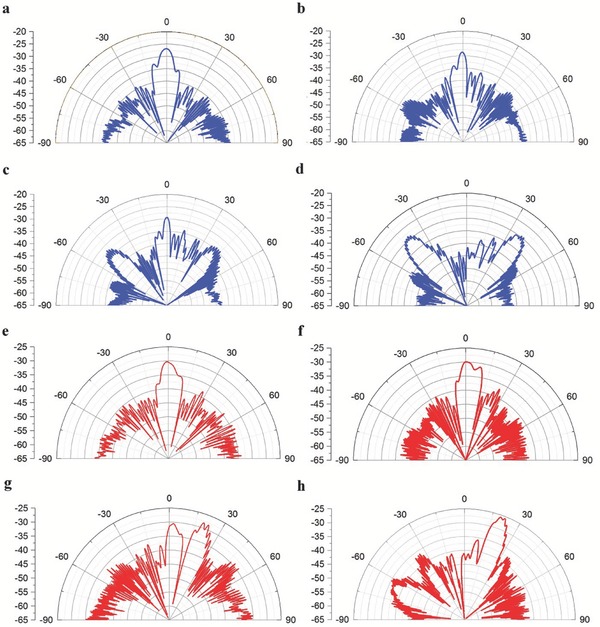
Experimental results of frequency coding metamaterial samples. a–d) Measured far‐field scattering patterns of the 1‐bit frequency coding metamaterials with the sequence of “0–0,” “0–1,” “0–0,” and “0–1” at a) *f* = 6 GHz, b) *f* = 8.5 GHz, c) *f* = 9.5 GHz, and d) *f* = 10.5 GHz. e–h) Measured far‐field scattering patterns of the 2‐bit frequency coding metamaterials with the sequence of “00–00,” “00–01,” “00–10,” “00–11,” “00–00,” and “00–01” at e) *f* = 6 GHz, f) *f* = 7.5 GHz, g) *f* = 9 GHz, and h) *f* = 10.5 GHz.

In experiments, the receiving antenna automatically recorded the electric field in the horizontal plane every 0.1° from 0° to 360°. For the 1‐bit frequency coding metamaterial sample, the measurement results showed that the incident plane waves were reflected back to the 0° direction by the metamaterial at the initial frequency *f*_0_ = 6 GHz (see **Figure** **8**a). Two symmetrical side lobes gradually appeared as the frequency increased, as shown in Figure 8b,c. Finally, the energy pointing to the *z*‐axis disappeared, and two side lobes evolved to be new beams at *f*_1_ = 10.5 GHz (see Figure 8d). For the 2‐bit frequency coding metamaterial sample, the measurement results showed that the incident plane waves were also normally reflected back by the metamaterial at the initial frequency *f*_0_ = 6 GHz (see Figure 8e). However, a side lobe pointing to the right side of the *z*‐axis gradually appeared as the frequency increased, as shown in Figure 8f,g. Finally, the energy pointing to the *z*‐axis disappeared, and the side lobe evolved to be a new main lobe at *f*_1_ = 10.5 GHz (see Figure 8h). The above results show good agreements with the simulation results illustrated in Figure 4a–d and Figure 5a–d, which prove to some extent the theoretical calculations and numerical simulation results.

## Conflict of Interest

The authors declare no conflict of interest.

## Supporting information

Supplementary
