# Supplementary material for: Controlling Energy Radiations of Electromagnetic Waves via Frequency Coding Metamaterials
Source: Adv Sci (Weinh). 2017 May 26;4(9):1700098. doi: 10.1002/advs.201700098 (PMC5604374; doi:10.1002/advs.201700098)
Supplement: Supplementary file 1 — Supplementary [file ADVS-4-na-s001.pdf]

Copyright WILEY-VCH Verlag GmbH & Co. KGaA, 69469 Weinheim, Germany, 2017.

Supporting Information

for *Adv. Sci.,* DOI: 10.1002/advs.201700098

Controlling Energy Radiations of Electromagnetic Waves via Frequency Coding Metamaterials

*Haotian Wu, Shuo Liu, Xiang Wan, Lei Zhang, Dan Wang, Lianlin Li, and Tie Jun Cui**

DOI: 10.1002/advs.201700098
Article type: Full Paper

Supporting Information

Controlling energy radiations of electromagnetic waves via frequency coding metamaterials

*Haotian Wu, Shuo Liu, Xiang Wan, Lei Zhang, Dan Wang, Lianlin Li and Tie Jun Cui**

Hao Tian Wu, Shuo Liu, Xiang Wan, Lei Zhang, Dan Wang, Prof. Tie Jun Cui*
State Key Laboratory of Millimeter Waves, Southeast University, Nanjing 210096, China

Prof. Lianlin Li
School of Electronic Engineering and Computer Sciences, Peking University, Beijing 100871, China

This file includes:
Supporting Text
Supporting Figs. S1

1. Far-field energy of 1-bit frequency coding metamaterials

We use the reflected electric field on the metamaterial $E(x)$ to calculate the far-field energy, as illustrated in Supporting **Figure S1**. Then the reflected energy by the metamaterial sample in the far distance is obtained as

$$E(r) = \int E(x)e^{j\omega t} \times e^{-j\frac{2\pi}{\lambda}r} dx \quad (16)$$

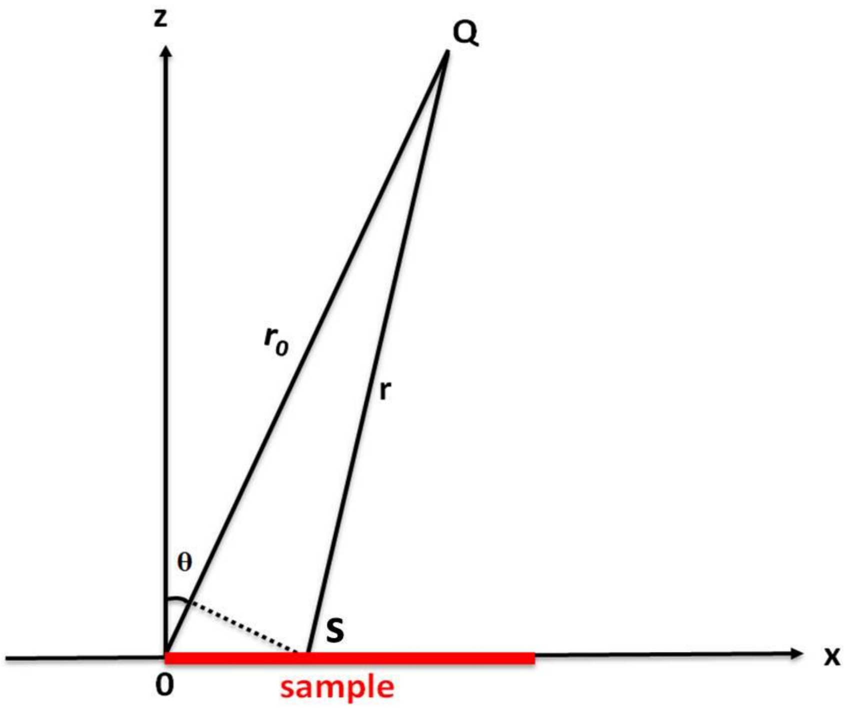

Supporting Figure S1. Schematic to calculate far-field energy of 1-bit frequency coding metamaterial.

The distance *r* between points *Q* and *S* can be rewritten as

$$r \approx r_0 - x\sin\theta \tag{17}$$

since *r* is much larger than *x*. Here, r_0 is the distance of observation point *Q* to the origin point, and θ is the angle between the observation point to the *z* axis, as shown in Supporting Figure S1. Hence $E(r)$ is expressed as

$$E(r) = \int E(x) \times e^{j\omega t} \times e^{-j\frac{2\pi}{\lambda}r} dx = e^{j\omega t} \times e^{-j\frac{2\pi}{\lambda}r_0} \int (E(x) \times e^{j2\pi\frac{\sin\theta}{\lambda}x}) dx \tag{18}$$

The phase distribution of 1-bit frequency coding metamaterial is 0, φ, 0, φ, 0, φ … with *N* periods. Thus $E(x)$ is expressed as:

$$E(x) = \sum_{n=0}^{2N-1} E_0 \times \Pi_D[x - nD] \times e^{j\frac{1}{2}[1-(-1)^n]\varphi} \tag{19}$$

where E_0 is the magnitude of $E(x)$, *D* is half length of a period, *N* is the number of periods, and

$$\Pi_D(x) = \begin{cases} 1 & -D/2 \le x \le D/2 \\ 0 & others \end{cases} \tag{20}$$

Then we denote

$$A = e^{j\omega t} \times e^{-j\frac{2\pi}{\lambda}r_0}$$
$$P = -2\pi \cdot \sin\theta / \lambda \quad (21)$$

Therefore, E(r) can be obtained as

$$E(r) = A \cdot \int (E(x) \times e^{-jPx})dx$$
$$= \text{A} \cdot \int \{\sum_{n=0}^{2N-1} E_0 \times \prod_D [x - nD] \times e^{j\frac{1}{2}[1-(-1)^n]\varphi} \times e^{-jPx}\}dx$$
$$= N \cdot D \cdot A \cdot E_0 \times s\text{in}\,c(\frac{PD}{2}) \times (1 + e^{j\varphi}) \quad (22)$$

When θ =0, we have P=-2π × sin θ/λ=0, and the corresponding far filed energy at angle θ=0 is proportional to

$$|E(r)|^2 = N^2 D^2 A^2 E_0^2 \times (1 + e^{j\varphi})^2 \quad (23)$$

where $N^2D^2A^2E^2$ is unchanged for a given sample. Hence the expression shows that the far-field energy at the angle θ=0 is proportional to $E^2|1+e^{j\varphi}|^2$ and φ is calculated from Equations (3), (5) and (6) in the main text as:

$$\varphi = 7\pi / 9 - [7\pi / 9 + (-\pi / 4.5) \times (f - f_0)] = 2\pi / 9 \times (f - f_0) \quad (22)$$

which increases from 0 to π as the frequency increases from 6 to 10.5GHz. As a consequence, the far-field energy at angle θ=0 should approach zero when the frequency is close to f_1=10.5GHz because $E^2|1+e^{j\varphi}|^2$ is approaching zero when the frequency is close to f_1=10.5GHz.

2. Non-periodic frequency sweeping metamaterials

The phase responses for 2-bit frequency coding units are

$$\varphi(f)^{'00-00'} \approx \frac{7\pi}{9}$$

$$\varphi(f)^{'00-01'} \approx \frac{7\pi}{9} - \frac{\pi}{9}(f - f_0)$$

$$\varphi(f)^{'00-10'} \approx \frac{7\pi}{9} - \frac{2\pi}{9}(f - f_0)$$

$$\varphi(f)^{'00-11'} \approx \frac{7\pi}{9} - \frac{3\pi}{9}(f - f_0) \quad (23)$$

Once the phase expressions are obtained, the phase differences of adjacent unit cells are calculated as

$$\varphi(f)^{'00-01'} - \varphi(f)^{'00-00'} \approx [\frac{7\pi}{9} - \frac{\pi}{9}(f - f_0)] - \frac{7\pi}{9} = -\frac{\pi}{9}(f - f_0)$$

$$\varphi(f)^{'00-10'} - \varphi(f)^{'00-01'} \approx [\frac{7\pi}{9} - \frac{2\pi}{7}(f - f_0)] - [\frac{7\pi}{9} - \frac{\pi}{9}(f - f_0)] = -\frac{\pi}{9}(f - f_0)$$

$$\varphi(f)^{'00-11'} - \varphi(f)^{'00-10'} \approx [\frac{7\pi}{9} - \frac{3\pi}{7}(f - f_0)] - [\frac{7\pi}{9} - \frac{2\pi}{9}(f - f_0)] = -\frac{\pi}{9}(f - f_0) \quad (24)$$

Note that phase differences between adjacent unit cells are the same and linearly proportional to the change of frequency f - f_0 in the operational band. From the generalized Snell's law, the deflected angle θ of the anomalous reflection beam is given by

$$\theta_r = \sin^{-1}[\frac{\lambda}{2\pi} \bullet \frac{d\varphi}{dx}] \quad (25)$$

Since $\frac{d\varphi}{dx} \approx \frac{\varphi^{'00-01'} - \varphi^{'00-00'}}{L}$ for approximation, in which L is the length of the super unit cell, and λ=$10^{-9}c/f$ (c=3×10^8m/s is the light speed in free space and the unit of f is GHz), the deflected angel θ_r is obtained as

$$\begin{aligned}\theta_r &= \sin^{-1}[\frac{\lambda}{2\pi} \bullet \frac{d\varphi}{dx}] \\ &\approx \sin^{-1}\{\frac{10^{-9} \times c}{2\pi f} \bullet [\frac{-\frac{\pi}{9}(f - f_0)}{0.006 \times 3}]\} \\ &= -\sin^{-1}[\frac{25}{27}(1 - \frac{f_0}{f})]\end{aligned} \quad (26)$$

Hence the reflection angle $|\theta_r|$ monotonically increases in the operational frequency band.
